# Case Report: Robotically Assisted Excision of Cystic Tumor Located in a Difficult to Access Area in the Liver

**DOI:** 10.3389/fsurg.2021.681012

**Published:** 2021-12-02

**Authors:** Evgeny Solomonov, Itamar Tzadok, Seema Biswas

**Affiliations:** ^1^Department of General and Hepatobiliary Surgery, Ziv Medical Center, Safed, Israel; ^2^Department of Transplantation, Rabin Medical Center and Tel Aviv University School of Medicine, Petah Tikva, Israel; ^3^Hepatobiliary Surgery Unit, Rabin Medical Center and Tel Aviv University School of Medicine, Petah Tikva, Israel

**Keywords:** liver, cyst, hepatopancreaticobiliary, robotic, minimally invasive

## Abstract

**Introduction:** Cystic liver lesions may be benign cysts, parasitic infestations, or malignant tumors requiring surgical resection. Hilar location and relation to major vasculature present challenges in conventional surgical access and resection.

**Materials and Methods:** We describe totally robotic excision of a cystadenoma in a 55-year-old woman without complication. Time points in the accompanying video (https://youtu.be/Tn_QPgpSHA4) are embedded within the text.

**Results:** Advantages of the robotic technique lie in overcoming the natural restriction of conventional laparoscopic instruments, easier repair, and control of intraoperative vascular injuries using EndoWrist^®^ instruments, ergonomic dissection close to major vasculature and reduced intraoperative blood loss as dissection is easier.

**Discussion:** Indications for robotic surgery included the large size of the cystic lesion, its intrahepatic location, and compression of the inferior vena cava (IVC) and right and middle hepatic veins. Had robotic removal of the lesion not been feasible, the entire lobe of the liver would have required resection.

## Introduction

Cystic liver lesions are frequently encountered in clinical practice. They represent a spectrum of differential diagnoses from benign cysts to parasitic infestations and malignant tumors (1, 2) that require surgical resection (3). In recent years in Beilinson Hospital, Rabin Medical Center, Israel, 300 liver resections have been performed. The ratio of open to laparoscopic resection is 1:10. The laparoscopic approach is preferred for non-malignant lesions (which require smaller resection margins), lesions measuring <5 cm, and lesions in the liver periphery—away from major liver vasculature. The laparoscopic approach was increasingly preferred by surgeons at Beilinson as they ascended the learning curve.

Since 2012, however, with the establishment of the Robotic Program for Liver Surgery at Beilinson Hospital, the Robotic Da Vinci Si System has revolutionized liver resection. The more favorable ergonomic features of the robotic approach further extended the selection of patients for minimally invasive surgery as major liver resections, right and left hepatectomy, right extended hepatectomy, radical cholecystectomy for cancer, and, crucially, central liver resections close to major vasculature—traditionally resected through the open approach—were performed safely and with greater ease. All liver cysts are now resected using robotic surgery, regardless of size, location, and complexity as experience of the robotic approach has widened the selection criteria.

In the case presented below, we explain our approach to robotic resection of liver cysts, in particular, cysts that present challenges in surgical access because of their location or relation to major vasculature. Such lesions, close to the hilum constitute at least 50% (6 out of 11) of all cyst excisions (at the time of writing) at Beilinson Hospital. These cysts would otherwise be resected through the open approach as the laparoscopic approach does not afford the same level of safety in access to the hilum and control of major liver vasculature. We explain the advantages of the robotic approach over both the open and laparoscopic approaches in terms of selection criteria, operative procedure, reduced postoperative pain, reduced opiate analgesia, reduced blood loss, and absence of need for blood transfusion.

## Materials and Methods

### Patient Data

The patient was a 55-year-old woman in good general health who undertakes daily sporting activity. She presented initially with epigastric pain with no history of jaundice, weight loss or previous surgery. Her comorbidities include well-controlled diabetes, hypertension, and hyperlipidemia. Liver function tests were slightly elevated (GGT, AST, and ALT) but tumor markers (CA19-9, CEA, and alpha-fetoprotein) were within normal limits (00:05–00:15). Echinoccocal serology was negative.

Ultrasonography showed a thin-walled cyst, 9 cm in diameter, located in segment 8, lying on the IVC inside the liver. No bile duct dilatation was seen. Tri-phasic computer tomography (CT) scan of the liver confirmed the 9 cm multi-lobulated cystic lesion in the liver. The IVC and right and middle hepatic veins were compressed (00:15–00:45) while the bifurcation of the portal vein abutted the cyst from above. At this stage, the differential diagnoses included biliary cyst adenoma and Echinococcal cyst. Although pre-operative radiology demonstrated no malignant features, until the time of surgery Echinococcal cyst remained a possibility, and the patient was, therefore, treated with a 2-month course of albendazole prior to surgery. Surgery was performed in July 2013. In the video we present a robotic approach for surgical excision by opening the liver through the parenchyma across the inter-lobar plane: the “open book” approach. This approach does not require entering the hilum and obviates the need to remove the gallbladder. Surgery was performed under general anesthesia. Deep muscle relaxation was important in order to obtain the largest available space (pneumoperitoneum) within which to work in the abdomen. The patient was placed in the supine position with 30-degree head up elevation and 15-degree left table tilt.

### The GelPOINT Device

The pneumoperitoneum was applied through a GelPOINT device (Applied Medical Ltd.) inserted through a small Pfannenstiel incision, safely under direct vision. The GelPOINT device contains a wound protector, silicone membrane, and trocars. The trocars are fixed in the membrane and may be used for additional access to the abdomen for the surgical assistant. The surgical assistant assists with additional traction on the tissues, inserts and withdraws sutures and performs suction. During the final step of the procedure the specimen—placed within the Endobag™ (Applied Medical Ltd.) device (Applied Medical Ltd.)—is extracted through the membrane of the GelPOINT device.

### Trocars

Four trocars are used for the robotic arm instruments and there is one trocar for the assistant. This permits access for additional manipulation of tissues by the assistant. Trocar placement was along a transverse line with maximum possible distance between the insertion points in order to prevent external arm collisions. The camera port was placed 2–3 cm lateral to and above the umbilicus, overlying the IVC as far as possible in front of an imaginary liver transection line—Cantlie's line. Division of the two liver lobes was planned from the diaphragmatic surface of the liver without entering the hilum.

### Procedure: (https://youtu.be/Tn_QPgpSHA4)

The lesion was covered by normal liver parenchyma and could not, therefore, be visualized directly within the abdominal cavity. Although, preoperative investigation suggested an absence of hydatid disease, the pericystic area, and operating field were covered with pads soaked in hypertonic saline. Under ultrasound guidance the cystic lesion was aspirated and injected with hypertonic saline. Clear, bile-stained fluid was aspirated. Aspiration reduced cyst tension (and may be helpful in cases of hydatid disease as this potentially reduces the risk of breach and dissemination of cyst contents). After exploration of the liver the falciform ligament was divided, reaching and dissecting the suprahepatic IVC and right and middle hepatic veins further defined by intraoperative ultrasound (01:16–01:20). Intraoperative ultrasound findings supported the preoperative diagnosis and assisted the localization of the middle hepatic vein on the surface of the liver. Using monopolar diathermy, the liver transection line was etched on the surface of the liver (00:58, 01:11–01:15). The third arm and the EndoWrist^®^ grasper were used for liver retraction. Access to the hepatic veins was facilitated by retracting the round ligament toward the left (01:06–01:10).

Next, adhesions to the gallbladder were divided in order to access the posterior surface of the right lobe of liver and the IVC (01:21–01:40). Transection of the liver parenchyma was commenced in the line drawn along the capsule of the liver. A harmonic scalpel was used to cut and seal small vessels. The waterjet dissector device was introduced by the assistant surgeon through the assistant port under the guidance of the console surgeon, and facilitated the identification of intrahepatic vascular structures—preventing unintentional injuries (01:49–02:25). One of the main branches of the middle hepatic vein pushed up and compressed by the tumor was encountered, identified, dissected, clipped using 10 mm endoclips, and cut (02:04–02:20).

Dissection to “open the liver” was begun with orientation of the surface of the cyst. The trunk of the middle hepatic vein was carefully approached and dissected away from the cyst, causing the cyst to emerge out of the liver parenchyma (02:24–02:30). In the same manner, small connecting branches were carefully ligated off the middle hepatic vein (02:44, 02:55, and 03:40–04:02). Dissection was continued around the cyst until the posterior wall had been exposed and anterior wall of the IVC, right hepatic vein and portal bifurcation were clearly visualized (03:22–04:48). Finally, the cyst was dissected off the IVC and right hepatic vein. Small tears were repaired with 6/0 polypropylene sutures. The cyst was placed within an Endobag™ (Applied Medical Ltd.) (05:28–05:35) and removed through the GelPOINT device. The cyst bed was lavaged using a suction-irrigation system (05:36–05:50). Small bile leaks were sutured using 5/0 polydioxanone sutures (05:55–06:09). The procedure was completed with the placement of a 10F Jackson-Pratt drain in the cyst bed (06:13–06:21). Total blood loss was <100 ml. No blood transfusion was required.

## Results

### Postoperative Recovery

The patient was able to walk on the first postoperative day. From the second day, she required no further opiate analgesia and was discharged from hospital on the fourth day with the Jackson-Pratt drain *in situ* for a further 5 weeks draining a low volume bile leak. In this time, she made a full recovery and resumed daily activities. After removal of the drain, she resumed swimming and sport. Histopathological examination confirmed biliary cystadenoma (mucinous cystic tumor) of the liver with no evidence of malignancy. Three months after surgery, follow-up CT scan showed no evidence of residual collection and the patient has good functional and cosmetic results (06:21–06:50). At follow up, more than 6 years later, the patient remains well.

## Discussion

With careful selection of cases, cystic, and malignant liver lesions may be safely excised laparoscopically (4, 5). Indeed, laparoscopic resections in difficult to access areas in the posterior liver segments and around central vasculature are well-documented. Montalti et al.'s metanalysis of robotic vs. laparoscopic liver resections reviewed articles published up to 2014. Laparoscopic resection was found to be associated with shorter operating times and less blood loss than the robotic approach (pneumoperitoneum plays a role in reducing blood loss as venous pressure becomes equalized). Clearly, the role of open, laparoscopic, and robotic approaches needs to be consolidated as expertise and experience with each approach accumulates (6–8). While a paucity of evidence precludes firm conclusions for now, robotic surgery does have obvious advantages where it may be performed safely in carefully selected cases. A recent meta-analysis from Singapore showed that robotic hepatectomy is associated with a shorter learning curve compared to the laparoscopic procedure (9, 10). Robotic techniques help to overcome the natural restriction of conventional laparoscopic instruments and allow safe and complete tumor removal. The EndoWrist^®^ instruments allow easy repair and control of intraoperative vascular injuries. Experience in major HPB surgery and laparoscopic liver resection is essential and cases such as this should be performed in centers with expertise in both. Indications for this particular procedure included the large size of the cystic lesion, its intrahepatic location, and compression of the IVC and right and middle hepatic veins. If this patient, had robotic removal of the lesion not been feasible, the entire lobe of the liver would have required resection. This has obvious implications for the morbidity to the patient. Preoperatively, the patient required a minimum of preparation (monitoring of blood sugars) with an enhanced recovery protocol. Postoperatively, she was able to mobilize on the first postoperative day and required no opiate analgesia from the second day, eating a full diet and mobilizing fully until discharge from hospital on the fourth postoperative day.

## Data Availability Statement

The original contributions presented in the study are included in the article/Supplementary Material, further inquiries can be directed to the corresponding author/s.

## Ethics Statement

Ethical review and approval was not required for the study on human participants in accordance with the local legislation and institutional requirements. The patients/participants provided their written informed consent to participate in this study. Written informed consent was obtained from the individual(s) for the publication of any potentially identifiable images or data included in this article.

## Author Contributions

ES, IT, and SB wrote and revised the manuscript. All authors contributed to the article and approved the submitted version.

## Conflict of Interest

The authors declare that the research was conducted in the absence of any commercial or financial relationships that could be construed as a potential conflict of interest.

## Publisher's Note

All claims expressed in this article are solely those of the authors and do not necessarily represent those of their affiliated organizations, or those of the publisher, the editors and the reviewers. Any product that may be evaluated in this article, or claim that may be made by its manufacturer, is not guaranteed or endorsed by the publisher.
