# Supplementary material for: Case Report: Robotically Assisted Excision of Cystic Tumor Located in a Difficult to Access Area in the Liver
Source: Front Surg. 2021 Dec 2;8:681012. doi: 10.3389/fsurg.2021.681012 (PMC8674714; doi:10.3389/fsurg.2021.681012)
Supplement: Supplementary file 1 [file Data_Sheet_1.ZIP › Data Sheet 1.PDF]

Consent Form for Case Reports

Case Report:

Robotically assisted excision of cystic tumor located in a difficult to access area in the liver

Principal Investigator: Dr Evgeny Solomonov, Rabin Medical Center, Tel: 054-7335762

You are being asked to consider allowing Dr. Evgeny Solomonov to use information about your cystic tumor excision surgery, to write what is called a case report. Case reports are typically used to share new unique information experienced by one patient during his/her clinical care that may be useful for other physicians and members of a health care team. A case report may be published (in print and/or via internet dissemination) for others to read, and/or presented at a conference. This form explains the purpose of this case report. Please read this form carefully and take your time to make your decision and ask any questions that you may have.

The purpose of this case report is to show and teach other hepatobiliary surgeons, the special surgical technique used during your procedure.

Your information being used for this case report includes demographic data (age, gender, medical history), radiology images (CT scans and Ultrasound), a video documenting the surgery, laboratory tests results from your pre-hospital evaluation, hospital stay and follow-up, and video segments not showing your face.

Dr. Evgeny Solomonov is obligated to protect your privacy and not disclose your personal information (information about you and your health that identifies you as an individual e.g. name, date of birth, medical record number). When the case report is published or presented, your identity will not be disclosed.

Although your personal information collected or obtained will be kept confidential and protected to the fullest extent of the law, there is a limited risk associated with this case report that could result in a loss of confidentiality by virtue of your unique experience.

You will not directly benefit from participating in this case report. The information that can be shared with other health care professionals, however, may improve the care that is received by others in the future.

Allowing your information to be used in this case report will not involve any additional costs to you. You will not receive any compensation.

Taking part in this case report is your choice (voluntary). You may choose not to take part or you may change your mind at any time. However, once the case report is written and published, it will not be possible for you to withdraw it. Your decision will not result in any penalty or loss of benefits to which you are entitled including the quality of care you receive.

You will be told about any new information relating to this case report that may affect you.

Your signature below means that you have read the above information about this Case Report and have had a chance to ask questions to help you understand how your information will be used and that you give permission to allow your information to be used in this case report.

If you have any questions please contact Dr Evgeny Solomonov at Tel: 054-7335762.

SUBJECT CONSENT TO PARTICIPATE

Case Report Title:

Robotically assisted excision of cystic tumor located in a difficult to access area in the liver

Name of Participant: __Bracha Bernstein__

Participant/Substitute decision-maker

By signing this form, I confirm that:

- The case report has been fully explained to me and all of my questions have been answered to my satisfaction
- I have been informed of the risks and benefits, if any, of allowing my information to be used in this case report
- I have been informed that I do not have to participate in this case report
- I have read each page of this form
- I authorize access to my personal health information (medical record) as explained in this form
- I have agreed to participate in this case report

Name of Participant/Substitute Decision-maker (print) Signature Date
