# Supplementary material for: Case Report: Robotically Assisted Excision of Cystic Tumor Located in a Difficult to Access Area in the Liver
Source: Front Surg. 2021 Dec 2;8:681012. doi: 10.3389/fsurg.2021.681012 (PMC8674714; doi:10.3389/fsurg.2021.681012)
Supplement: Supplementary file 1 [file Data_Sheet_1.ZIP › Data Sheet 2.PDF]

<u>טופס הסכמה מדעת- שימוש במידע רפואי שנאסף במהלך פעולה</u>

<u>הצגת מקרה:</u> **ניתוח באמצעות רובוט להסרת ציסטה מהכבד באזור אנטומי לא נגיש**
Robotically Assisted Excision of Cystic Tumor Located in a Difficult to Access Area in the Liver

<u>חוקר ראשי:</u> ד"ר יבגני סולומונוב, מרכז רפואי רבין, טל' 054-7335762

בטופס זה, נבקש ממך רשות בשם **ד"ר יבגני סולומונוב** לעשות שימוש במידע שנאסף במהלך האשפוז והניתוח שעברת להסרת ציסטה בכבד, לשם כתיבה של מאמר מסוג "הצגת מקרה". מאמר "הצגת מקרה" הוא דרך המשמשת אנשי מקצוע בתחום הרפואה לחלוק מידע רפואי ייחודי על הטיפול (תרופתי, כירורגי וכו') שעבר מטופל יחיד. מאמר "הצגת מקרה" יכול להיות מפורסם בעיתון מדעי/רפואי מודפס או באינטרנט כדי שאנשי מקצוע אחרים בתחום יוכלו לקרוא אותו, או להיות מוצג בכנס מדעי/רפואי לאנשי מקצוע בתחום. בטופס זה, נסביר את מטרת מאמר מסוג "הצגת המקרה" עבור הטיפול הייחודי שעברת. אנא הקדש את כל הזמן הנחוץ כדי לקרוא את הטופס היטב ולהחליט האם לתת הסכמה. הרגש/י חופשי/ה להפנות שאלות במידה והן עולות לחוקר הראשי בנוגע לתכני המסמך.

מטרת מאמר "הצגת המקרה" המדובר היא לחשוף כירורגים בתחום ההפטו-ביליארי לטכניקת הניתוח הייחודית באמצעות רובוט, כפי שבוצעה בטיפולך בתאריך 21.7.2013 במרכז רפואי רבין.

נבקש ממך הסכמה לעשות שימוש במידע האישי הבא: פרטים דמוגרפיים כלליים (גיל, מין, מחלות רקע), צילומי בדיקות דימות (בדיקות CT וסונוגרפיה), סרט וידאו מזמן הניתוח, תוצאות בדיקות מעבדה מזמן האשפוז שקדם לניתוח ולאחריו, ותמונות סטילז שאינן חושפות את הפנים.

ד"ר יבגני סולומונוב מתחייב להגן על פרטיותך, ולא לחשוף מידע אישי (כלומר, מידע עליך ועל בריאותך שמאפשר זיהוי עתידי שלך, לרבות- שם, תאריך לידה, מס' רשומה רפואית). כאשר מאמר "הצגת המקרה" יפורסם או יוצג, זהותך תשמר חסויה.

למרות שהמידע האישי שלך יישמר חסוי ומוגן לפי החוק, קיים סיכון מינימלי לאובדן החיסיון הרפואי בשל ייחודה של הפעולה הרפואית שעברת.

פרסום מאמר "הצגת מקרה" לא תביא לך רווח אישי ישיר. יחד עם זאת, המידע שיפורסם ב"הצגת המקרה" יוכל להגיע למומחים נוספים בתחום, ולהוביל לשיפור בטיפול שיעברו מטופלים אחרים בעתיד.

מתן הסכמה לשימוש במידע הרפואי למטרת "הצגת מקרה" אינה כרוכה בתשלום נוסף מצדך, או בקבלה של פיצויים.

השתתפות במאמר מסוג "הצגת מקרה" היא מבחירתך החופשית בלבד. ביכולתך לבחור לא להסכים להשתתף במאמר "הצגת מקרה", או לשנות את דעתך בכל שלב בעתיד. יחד עם זאת, מרגע שפורסם מאמר "הצגת מקרה" לא יהיה ניתן להסיר אותו מהעיתון המדעי בו הוא פורסם. החלטתך לתת או לא לתת הסכמה מדעת לא תפגום באיכות הטיפול שמגיעה לך, ולא תגרור קנסות או ענישה.

ניידע אותך בדבר כל שינוי או מידע חדש שנוסיף למאמר "הצגת המקרה".

בחתימתך בסוף מסמך זה, את/ה מצהיר/ה כי קראת את הטופס בשלמותו, וקיבלת מידע על מאמר מסוג "הצגת מקרה", וכי ניתנה לך הזדמנות לשאול שאלות לשם הבהרת המידע שנמסר במסמך זה. כמו כן, בחתימתך את/ה נותנ/ת רשות לעשות שימוש במידע הרפואי לשם כתיבת מאמר "הצגת מקרה".

במידה ויש לך שאלות נוספות, ניתן ליצור קשר עם **ד"ר יבגני סולומונוב** לשם קבלת הסברים נוספים בטל' 054-7335762.

הסכמת המטופל להשתתפות במאמר מסוג "הצגת מקרה"

הצגת מקרה: ניתוח באמצעות רובוט להסרת ציסטה מהכבד באזור אנטומי לא נגיש
Robotically Assisted Excision of Cystic Tumor Located in a Difficult to Access Area in the Liver

שם המטופל: הרנשטיין ברכה

הצהרת המטופל / אפוטרופוס:

בחתימה עם טופס זה, אני מצהיר כי:

- מטרותיו של מאמר "הצגת המקרה" הוסברו לי במלואן, וקיבלתי מענה הולם לכל שאלותיי בנושא.
- יידעו אותי בדבר הסיכונים והתועלת, אם יתממשו, הכרוכים בכך שייעשה שימוש במידע הרפואי שלי לטובת "הצגת המקרה".
- יידעו אותי כי אני לא חייב לתת את המידע הרפואי שלי לטובת מאמר מסוג "הצגת מקרה".
- קראתי את כל העמודים בטופס ההסכמה מדעת (ע"מ 1 ו-2).
- אני מאשר גישה לרשומות הרפואיות שלי, כפי שמפורט בטופס ההסכמה מדעת.
- אני נותן הסכמתי להשתתף במאמר "הצגת מקרה".

הרנשטיין ברכה	[illegible]	10.12.17
שם המטופל / אפוטרופוס	חתימה	תאריך

ד"ר [illegible]	[illegible]	10.12.17
שם החוקר הראשי	חתימה	תאריך
